# Canadian Society of Allergy and Clinical Immunology position statement: panel testing for food allergies

**DOI:** 10.1186/s13223-024-00937-0

**Published:** 2024-11-29

**Authors:** Abdulrahman Al Ghamdi, Elissa M. Abrams, Stuart Carr, Mariam A. Hanna, Sari M. Herman, Elana Lavine, Harold Kim, Timothy K. Vander Leek, Douglas P. Mack

**Affiliations:** 1 Division of Allergy and Immunology, Department of Pediatrics, University of Toronto, Toronto, Canada; 2 Department of Pediatrics, King Faisal Specialist Hospital and Research Center, Jeddah, Saudi Arabia; 3 Department of Pediatrics, Section of Allergy and Clinical Immunology, University of Manitoba, Winnipeg, Canada; 4 Department of Pediatrics, Division of Allergy, University of British Columbia, Vancouver, Canada; 5 Snö Asthma & Allergy, Abu Dhabi, United Arab Emirates; 6 McMaster University, Hamilton, ON Canada; 7 Department of Medicine, Temerty Faculty of Medicine, University of Toronto, Toronto, Canada; 8 Department of Pediatrics, University of Toronto & Humber River Hospital, Toronto, ON Canada; 9 Western University, London, ON Canada; 10 Canada and McMaster University, Hamilton, ON Canada; 11 Department of Pediatrics, Stollery Children’s Hospital, University of Alberta, Edmonton, AB Canada; 12 Department of Pediatrics, University of British Columbia, Vancouver, Canada; 13 Department of Pediatrics, McMaster University, Hamilton, ON Canada; 14 Halton Pediatric Allergy, 5500 North Service Road, Suite 106, Burlington, ON L7L 6W6 Canada

**Keywords:** Food allergy, Screening test, Oral food challenge, Harm reduction, Prevention of allergy, Diagnosis, Test limitations

## Abstract

This position statement addresses the critical concerns and recommended practices surrounding the use of panel food testing for diagnosing food allergies. Food allergies are a significant public health concern, and the misdiagnosis of food allergies remains a prevalent concern, made worse by the ongoing use of panel food testing. The practice of screening patients for multiple food allergens, regardless of clinical relevance, is commonly referred to as “panel food testing.” Fundamentally, a panel food test is not simply a single test; a panel food test is a series of several distinct tests for multiple foods, each with its own variable predictive value. These tests have not been adequately validated as screening tests and carry a considerable false positive rate. The resulting false diagnoses lead to unnecessary dietary restrictions, increased healthcare costs, and significant psychosocial distress for patients and their families.

This statement calls for the judicious use of food allergy testing, understanding the limitations of these tests and the potential for harm when panel food tests are used. Due to the potential for significant long-term harm, panel food testing for foods that have not led to clinical reaction should be actively discouraged. By limiting panel food testing and incorporating validated practices such as oral food challenges, allergists and other clinicians can mitigate the risks associated with misdiagnosis and ensure a rational, patient-centred approach to food allergy testing.

Food allergies represent a significant public health concern, with approximately 5–10% of the Canadian population reporting a food allergy diagnosis [1]. In recent years, the importance of early introduction of priority allergens, such as peanuts, to prevent IgE-mediated food allergy in infants has been reinforced through large-scale randomized controlled trials and international guidelines. [2] Furthermore, advances in food allergy treatment, including immunotherapies, promise to improve long-term outcomes.

Although these advances in the primary prevention and treatment of food allergies are encouraging, misdiagnosis of food allergies remains problematic. While diagnostic allergy tests (skin prick testing, serum food-specific IgE) have been widely available and commonly used for decades, they have not been validated as screening tools for food allergies, and their use may lead to false positive diagnoses and longer-term impairment of quality of life [3]. Although more specific diagnostic tests, such as basophil activation testing (BAT) or food component IgE testing, are being developed, they are not routinely available or standardized for all populations [3]. Oral food challenges (OFCs) continue to be the gold standard for diagnosing food allergy, though access remains restricted due to physician, patient, and system factors across Canada [4].

*The practice of screening at-risk patients for multiple food allergens, regardless of clinical relevance, is commonly referred to as “panel food testing”* Such panel testing may cause harm through false diagnoses, unnecessary costs, and patient and parent/caregiver anxiety. (Table 1) The resulting delay in the introduction and unnecessary avoidance of priority food allergens may cause iatrogenic food allergy, the very patient outcome we strive to reduce [5]. Moreover, the management of falsely diagnosed food allergies may lead to unnecessary and costly avoidance strategies, additional investigations, and treatment.

**Table 1:** The potential impacts of panel food testing

Negative impacts	Positive impacts
Loss of opportunity for tolerance acquisition/primary prevention through unnecessary avoidance	Prevention of index reaction
Loss of acquired tolerance through unnecessary avoidance	Potential reduction in parental anxiety
Psychological impact of food allergy diagnosis	
Cost of food allergy avoidance strategies and alternative, allergy-safe foods	
Medication costs, including antihistamines, epinephrine and immunotherapy options	
Personal and societal cost of food allergy diagnostic and medical management	
Social barriers, including schools, daycares, camps and family	
Nutritional implications include reduced dietary diversity, malnutrition, impaired growth	
Unnecessary testing costs, consultation costs and oral food challenge costs in an underserviced and overburdened health care system	

This statement evaluates the practice of panel food testing and provides recommendations for optimizing relevant food allergy testing.

For the purposes of this manuscript, we define ‘*sensitization’* as having evidence of food-specific IgE with or without the presence of clinical hypersensitivity, while the terms ‘*food* allergy/*allergic’* or ‘*true food allergy’* refer to individuals with both evidence of food-specific IgE and clinical hypersensitivity to that food. (Fig. 1).

**Fig. 1:**
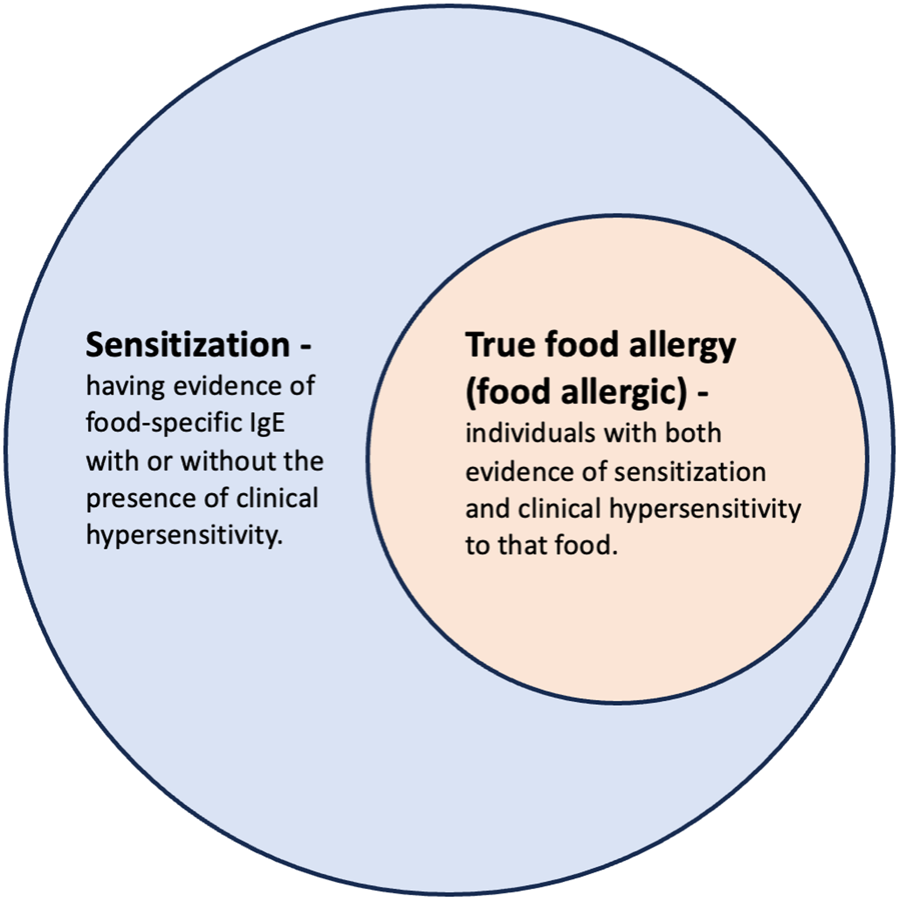
Sensitization versus True Food Allergy

## Are food panel tests validated screening tests?

Wilson and Jungner first proposed the rigorous definition of screening, with principles providing a structure to ensure accountability for the application of screening tests [6]. An update in 2018 highlighted the importance of establishing the accuracy, reliability, and reproducibility of screening tests, ensuring high sensitivity, specificity and predictive values in all relevant target populations [7]. The authors also emphasized the importance of having accurate and timely postscreening diagnostic and treatment options available before widespread screening is implemented. These criteria further prioritized the consideration of the balance between benefits and risks, including overdiagnosis and overtreatment.

Fundamentally, a panel food test is not simply one test; it is a series of several distinct tests, each for a separate food and each with its own variable predictive value, dependent on the patient's history and clinical characteristics. None of these individual tests can be characterized as highly predictive in previously nonreactive patients. One study determined that the positive predictive value of food panel testing was only 2.2% in their referral population [8]. Therefore, using these tests inappropriately as “screening tests” ultimately leads to false diagnoses in many patients.

Unfortunately, there are few high-quality data to provide robust validation of individual or panel food testing as a screening tool for food allergies for most foods. A recent Joint Task Force practice parameter for the diagnosis of peanut allergy recognized the reasonable utility of peanut testing in populations that *have experienced a previous allergy reaction to peanuts*. [9] This is not surprising since the pretest probability in this reactive population is relatively high. However, this parameter also acknowledged the significant difficulty in interpreting these tests when the pretest probability of allergy is lower. In general, when screening tests are used to *predict* the presence of food allergy, the pretest probability is far lower than when a patient has previously reacted to the food.

Although reports of clinical experience and potential predictive values have been published for other foods for very specific populations, including some tree nuts, the data are not robust and do not apply to all patient populations. A recent systematic review revealed major limitations of current testing strategies [10]. This review noted substantial variability in specificity and noted limited data sources for meta-analysis, with significant heterogeneity based on age and geographic location. Moreover, for most foods, much of the data on testing were retrospective, with no prospective randomized controlled trials identified. Furthermore, the oral food challenge (OFC) was not universally used as a standard for diagnosis in many of the source articles, undermining this review [10]. Notably, the validity of testing in infants has not been well established, especially before the introduction of allergenic food.

The real-world practical shortcomings of these approaches have been demonstrated in several articles. Couch et al. published an evaluation of tree nut OFCs in patients with a mean age of 4.5 years. [11] A total of 76% of patients with a history of tree nut allergy proved tolerant of unrelated nuts to which they were sensitized. Ninety-one percent of patients with sensitization, without prior reaction or ingestion, passed the OFC. Strikingly, in patients with peanut allergies and tree nut sensitization, the OFC success rate for tree nuts was 96%. In another series of more than 1000 infants with atopic dermatitis, panels of food-specific IgE were performed [12]. In this large series, even with a cutoff of 100 kU/L, the probability of having a true food allergy did not reach 50% for most foods. The low specificity of these tests can be attributed to the relatively large number of people who are sensitized to certain foods (i.e., have detectable specific IgE) but who are not truly allergic and do not have adverse reactions when consuming these foods in their diet. It is well established that sensitization is essential but not sufficient to cause food allergies, and epidemiological studies from North America and Europe have consistently demonstrated that the prevalence of food sensitization is much greater than that of true food allergies. [13–15]

Furthermore, in agreement with Wilson and Jungner, access to postscreening diagnostic options is critical to implementing a screening test [6, 7]. In an ideal situation, each positive test is followed up in a timely manner with an OFC. However, the lack of access to OFCs has been well documented. An American publication noted that only 17% of allergists offer more than 10 OFCs per month, with 35.5% of allergists never offering OFCs to infants under 1 year of age [16]. Similarly, Canadian data are alarming, with nearly 20% of allergists not offering OFCs and nearly 50% not offering them to infants [4]. Systemic barriers to accessing OFCs were well documented in both publications. Regardless of access to the initial diagnostic screen, there is simply no ability to provide timely diagnostic certainty for these sensitized patients.

Unfortunately, implementing any food allergy screening recommendations has been associated with a “screening creep” with increased tests in the broader population. A recent evaluation of infant food testing demonstrated an increased number of patients tested following the 2017 NIAID guidelines for the prevention of peanut allergy, even among low-risk infants. [17] Most concerning, despite the guidelines specifying testing only for peanuts, the median number of foods tested was 10. Not surprisingly, few infants in this group of previously nonreactive or unexposed infants were offered an OFC for any foods that tested positive. [18]

Finally, screening for food allergies is not cost-effective at the population level. A recent economic analysis demonstrated an excess cost of over one billion dollars for peanut allergy screening in infants [19]. Although there are limited available data for other foods, the cost of screening panel testing is particularly concerning, given the propensity for expanding food testing [20].

It is difficult to apply the rigorous principles of screening for disease to allergy tests, whether a skin prick test (SPT) or serum-specific IgE test is used. Recognizing that each food test in a panel represents an individual, unique test is fundamental to understanding these shortcomings. While these tests can be helpful from a diagnostic perspective in patients with a clear and concerning clinical history, they fall short of the requirements to provide accurate screening.

## In an allergic population, is there potential harm from panel food testing?

In patients who are known to be atopic, there are inherent risks to panel testing for food.

allergy. The most obvious is the risk of false positive or clinically irrelevant results and, thus, an inappropriate food allergy diagnosis, especially if OFCs are not offered, as both skin prick testing and food-specific IgE testing lack specificity [21]. Even in patients with a history consistent with food allergy, the positive predictive value of skin prick testing or specific IgE varies depending on the patient’s medical history, the cutoff used and the potential allergen. [8, 22, 23]

Early and regular allergen exposure is critical to the development and maintenance of oral tolerance, and inappropriate allergy testing and resultant false food allergy diagnoses can lead to unnecessary food avoidance, which has significant potential to cause loss of tolerance (or failure to develop tolerance) and the subsequent development of true food allergy. In this manner, these well-intentioned but misguided tests may act as a self-fulfilling prophecy, contributing to the development of food allergies where one previously did not exist, a form of *iatrogenic food allergy*. [24] This harm is not just theoretical, especially in young children and infants, as many reports have demonstrated that atopic patients are more likely to develop a food allergy following delayed introduction or prolonged avoidance of food [2, 25, 26]. This is, perhaps, even more of a risk if a person is already sensitized to a potential allergen, as seen in a report by Flinterman et al., where all 11 of their patients sensitized to cow milk developed true cow milk allergy after a median time of avoidance of 2.3 years with no significant improvement in their atopic dermatitis, which was the initial reason for avoidance. [27] Furthermore, the Pronuts study confirmed the age-based development of tree nut and seed allergies among children who avoided these food allergens unnecessarily.

Similarly, the Learning Early About Peanut (LEAP) study demonstrated that infants who were sensitized to peanuts were more likely to benefit from early introduction. Fundamentally, the LEAP study demonstrated that the avoidance of food such as peanuts for any reason, including as a result of false positive tests, contributed to the development of true and potentially life-long allergies [28]. Critically, recent data suggest that the benefit of early peanut introduction is markedly age-dependent and that delays can lead to peanut allergy development. As clinicians, the imperative to “do no harm” should govern our approach to these clinical scenarios, and generating a false diagnosis causes definitive harm to that patient.

A diagnosis of food allergy is also not without risk to patients’ psychological health, as it has been linked to increased anxiety and poorer quality of life outcomes. [29, 30] According to a recent review, children and adolescents with food allergies are more likely to develop symptoms of anxiety, depression, and feeding and eating disorders such as anorexia nervosa [31]. These negative effects are not only limited to the patients themselves but can also affect their families, particularly their primary caregivers. Children and adolescents with food allergies are also more likely to be victims of bullying, with their food allergies often being used in the process of bullying [32].

Another potential negative health impact on patients with a misdiagnosis of food allergy is malnutrition, particularly in children with multiple food allergies. Reviews of the numerous studies on the nutritional impact of food allergies have shown that children with food allergies are more likely to be smaller than their nonallergic peers. They may also develop micronutrient deficiencies, feeding aversion, or feeding difficulty [33, 34] In some cases, severe malnutrition may result from excessive avoidance, as reported in a patient with a false diagnosis of food allergy [35].

The financial impact of a diagnosis of food allergy is substantial. While the cost of specialist allergy consultations is typically covered through provincial health insurance plans for most patients in Canada, other costs, such as specific IgE testing, epinephrine autoinjectors, travel costs, work absenteeism, and treatments, are not universally covered nationwide. Avoiding a food allergen can also be costly, as most allergen substitute foods (e.g., hypoallergenic formula) are more expensive than their allergenic counterparts. This cost is more pronounced in patients diagnosed with multiple food allergies who require even more restrictive diets. Beyond these immediate medical costs, a 2019 paper evaluating the cost of food allergy reviewed 11 studies from the USA and Europe and showed that the household-level opportunity cost of food allergy had the highest economic burden on families with a member who has a food allergy [36]. They defined opportunity cost as loss of potential earnings that result from food allergy, of which some examples included decreased labour productivity, loss of leisure activity, and increased time spent on food allergy-related household tasks and information seeking [36]. These indirect costs may be underestimated by healthcare providers but are a significant cause of lost income for families dealing with food allergies.

Additionally, many false diagnoses require otherwise unnecessary OFCs, which increase health care to an overburdened and underserviced system. Preemptive testing for peanut allergy in infants in the US has been modelled to cost between $654 million and $2.46 billion and result in an excess of 8,000 peanut-allergic children, likely related to delayed administration and false positive testing [19].^.^ Importantly, in the age of active therapies such as oral immunotherapy, false-positive testing also leads to unnecessarily treated patients, with a significant cost to the patient and the healthcare system [24, 37]. These excess healthcare costs are not sustainable in our current health system.

Other less significant potential negative impacts of allergic panel testing include the tests' physical characteristics. Skin prick testing can be uncomfortable, anxiety-inducing, and time-consuming, while specific IgE testing is expensive and requires venipuncture, which may be difficult in younger children.

Panel testing for food allergies is not a harmless procedure and has potential negative physical, psychological, and financial impacts. As substitute decision-makers, parents and clinicians must act in the child’s best interests to obtain as accurate a diagnosis as possible while mitigating harm to the patient.

## Is there a potential benefit from food allergy panel testing in patients who have not shown typical food allergy symptoms?

Some clinicians consider testing with the intent of diagnosing an allergy before developing symptoms. Indeed, the NIAID peanut allergy prevention guideline addendum from 2017 suggested testing before introducing peanuts to infants with severe atopic dermatitis or egg allergy [38]. While precautionary testing in this situation may identify patients who may develop peanut allergy prior to a clinical reaction, as we outline in the previous question, the harms of this approach and the potential for false positive tests can be substantial. In fact, the NIAID guidelines largely stand alone in the international community, where food introduction is safely prioritized over preemptive testing [28]. Furthermore, there has been no validation of this screening approach in this population. No guidelines endorse widespread testing of foods as part of a panel, in an at-risk population or otherwise.

Another rationale offered for panel testing by some providers is to identify potential food triggers for atopic conditions such as severe atopic dermatitis and eosinophilic esophagitis. Although testing in these settings is not the focus of this statement, the evidence for such practice is extremely limited and not supported by the current evidence, and the relationship between food-specific IgE levels and non-IgE-mediated atopic disease has not been established. [39, 40]

Some physicians may perform such testing in an attempt to rule out a food allergy as the cause of a patient’s nonspecific symptoms or as a method to relieve patient or parental anxiety about food introduction. While there is a potential benefit if testing proves negative, this approach may paradoxically lead to worsening anxiety or unnecessary avoidance if the testing is unexpectedly positive with irrelevant sensitization, and families have delays in further diagnostic confirmation [41]. If such testing is performed despite the strong recommendations against doing so, there must be clear communication to the patient about the reason for testing, along with a plan to proceed with more definitive testing (i.e., oral food challenge) should the testing be positive. Regardless, there is no evidence demonstrating that this approach to addressing parental anxiety is beneficial.

Finally, positive food test results on panel tests may lead to the false attribution of a patient’s symptoms to food allergy, misleading patients and their caregivers, and resulting in delays in the diagnosis of other more relevant medical conditions.

## What are the alternatives to food allergy panel testing?

Appropriate alternatives may be recommended depending on the reason for panel testing.

Given the weak and contradictory evidence, in the case of patients with other atopic conditions, such as atopic dermatitis, and no symptoms of immediate food allergy, we recommend against food allergy testing, panels or other methods of managing the underlying atopic disease, as food avoidance alone rarely leads to improvement in the disease.

In cases of suspected food allergy, OFCs should be offered for patients with positive skin or specific IgE test results but no clear history of significant reaction when ingesting food. As noted above, OFCs remain the gold standard for diagnosing food allergies [42].

For infants or other patients who have never ingested a particular food, we recommend home introduction of the food before any testing and limiting testing only to patients who have symptoms with ingestion. Families can be reassured that the probability of severe reactions with infant food introduction is exceedingly low, with most reactions being mild and self-resolving [2, 28, 43]. In cases where parents are very hesitant about introduction at home, in-office introduction may also be an option to help alleviate anxiety and provide reassurance. If necessary, access to a food allergy counsellor may also be beneficial.

Whatever the decision that is made with the patient or their parents, clear communication and explanation of the limitations of testing and its potential harms and intended benefits should be sought in all encounters [44].^.^

## Summary and recommendations

**Fig. 2:**
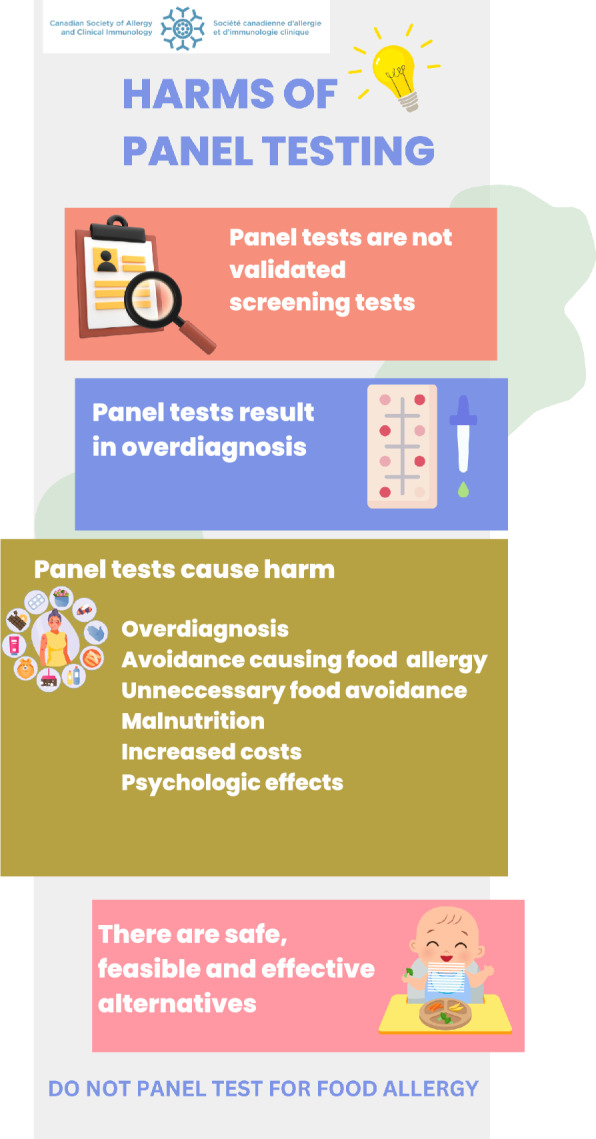
Infographic for patient and clinician education regarding the harms of panel food testing

An infographic has been developed to assist patient and physician education (Fig. 2).

- 1. Due to the potential for significant long-term harm, panel food testing for foods that have not been eaten should be actively discouraged.
- 2. Testing should not be performed for foods that are being consumed on a regular basis without immediate symptoms consistent with IgE-mediated allergy.
- 3. If such testing is performed despite the strong recommendations against doing so, there must be clear communication to the patient about the reason for testing, along with a plan to proceed with more definitive testing (i.e., oral food challenge) should the testing be positive.
- 4. Increase access to and support for oral food challenges.

## Conclusion

Although the practice of indiscriminate food testing is discouraged, it is essential that allergists revisit the fundamental principles of screening and encourage dialogue surrounding the significant limitations of our current testing approaches. Panel food testing can actively cause harm to patients, and allergists must remain critical of this outdated practice.

## Data Availability

No datasets were generated or analysed during the current study.
